# Mechanical load-induced H_2_S production by periodontal ligament stem cells activates M1 macrophages to promote bone remodeling and tooth movement via STAT1

**DOI:** 10.1186/s13287-020-01607-9

**Published:** 2020-03-13

**Authors:** Danqing He, Fuliang Liu, Shengjie Cui, Nan Jiang, Huajie Yu, Yanheng Zhou, Yan Liu, Xiaoxing Kou

**Affiliations:** 1 Department of Orthodontics, Peking University School and Hospital of Stomatology, 22# Zhongguancun South Avenue, Haidian District, Beijing, 100081 China; 2 National Engineering Laboratory for Digital and Material Technology of Stomatology, 22# Zhongguancun South Avenue, Haidian District, Beijing, 100081 China; 3 Beijing Key Laboratory of Digital Stomatology, 22# Zhongguancun South Avenue, Haidian District, Beijing, 100081 China; 4 Department of Orthodontics, ShenZhen Clinic, Sunny Dental Group, #2388 Houhai avenue, Nanshan District, Shenzhen, 518100 China; 5 Central laboratory, Peking University School and Hospital of Stomatology, 22# Zhongguancun South Avenue, Haidian District, Beijing, 100081 China; 6 Fourth Division, Peking University School and Hospital of Stomatology, No. 41 Dongsuhuan Road, Chaoyang District, Beijing, 100025 China; 7 South China Center of Craniofacial Stem Cell Research, Hospital of Stomatology, Sun Yat-sen University, 74 Zhongshan 2Rd, Guangzhou, 510080 China

**Keywords:** Stem cells, Macrophage polarization, Hydrogen sulfide, Bone remodeling, Mechanical load, Cell signaling

## Abstract

**Background:**

Tooth movement is a unique bone remodeling process induced by mechanical stimulation. Macrophages are important in mediating inflammatory processes during mechanical load-induced tooth movement. However, how macrophages are regulated under mechanical stimulation remains unclear. Mesenchymal stem cells (MSCs) can modulate macrophage polarization during bone remodeling. Hydrogen sulfide (H_2_S) can be produced by MSCs and have been linked to bone homeostasis. Therefore, this study aimed to investigate whether H_2_S contributed to periodontal ligament stem cell (PDLSC)-regulated macrophage polarization and bone remodeling under mechanical stimulation.

**Methods:**

An experimental mechanical load-induced tooth movement animal model was established. Changes in cystathionine-β-synthase (CBS), markers of M1/M2 macrophages, tooth movement distance, and the number of osteoclasts were examined. The conditioned medium of PDLSCs with or without mechanical loading was utilized to treat THP-1 derived macrophages for 24 h to further investigate the effect of PDLSCs on macrophage polarization. Different treatments with H_2_S donor, CBS inhibitor, or the inhibitor of STAT1 were used to investigate the related mechanism. Markers of M1/M2 polarization and STAT1 pathway expression were evaluated in macrophages.

**Results:**

Mechanical load promoted tooth movement and increased the number of M1-like macrophages, M1-associated pro-inflammatory cytokines, and the expression of CBS on the compression side of the periodontal ligament. The injection of CBS inhibitor or H_2_S donor could further repress or increase the number of M1-like macrophages, tartrate-resistant acid phosphatase-positive osteoclasts and the distance of tooth movement. Mechanistically, load-induced PDLSCs enhanced H_2_S production, which increased the expression of M1-associated cytokines in macrophages. These effects could be blocked by the administration of CBS inhibitor. Moreover, load-induced H_2_S steered M1 macrophage polarization via the STAT1 signaling pathway.

**Conclusions:**

These data suggest a novel mechanism indicating that mechanical load-stimulated PDLSCs produce H_2_S to polarize macrophages toward the M1 phenotype via the STAT1 signaling pathway, which contributes to bone remodeling and tooth movement process. These results provide new insights into the role of PDLSCs in regulating macrophage polarization and mediating bone remodeling under mechanical stimulation, and indicate that appropriate H_2_S supplementation may accelerate tooth movement.

**Electronic supplementary material:**

**Supplementary information** accompanies this paper at 10.1186/s13287-020-01607-9.

## Background

Mechanical stimulation is important in promoting tissue development and maintaining tissue homeostasis [1–3]. Mechanical stimuli can regulate communications among different cell types and between the cells and tissue microenvironment [4, 5]. Among all the systems of our body, the skeletal system, including alveolar bone, specifically responds to changes in mechanical load [6, 7]. Mechanical load can enhance the expressions of inflammatory cytokines, chemokines, and β-2 adrenergic receptor in the surrounding tissues of alveolar bone and activate immune cells such as macrophages and T lymphocytes, which may influence alveolar bone remodeling [8–12]. However, the detailed mechanism of how mechanical stimuli modulate alveolar bone remodeling remains unclear.

Tooth movement is a unique bone remodeling process induced by mechanical stimulation. During this process, aseptic inflammation develops on the compression side of periodontal ligament, which leads to bone resorption on the compression side and bone formation on the tension side [8, 13]. Macrophages, the main immune cells and the precursors of osteoclasts, play a critical role in developing inflammation and mediating bone remodeling during tooth movement [14, 15]. Macrophages could be polarized into different phenotypes under different environmental elements and perform different functions [16]. “Inflammatory” classically activated M1 polarization is mainly induced by interferon (IFN)-γ or lipopolysaccharides and mediates the inflammatory process by producing inflammatory elements such as tumor necrosis factor (TNF-α) and nitric oxide (NO); furthermore, M2 polarization is mainly activated by interleukin (IL)-4 or IL-13 and plays an important role in tissue remodeling by producing IL-10 and arginase I [17–19]. Previously, we observed that M1 macrophage polarization contributes to bone remodeling and root resorption during tooth movement [10, 20]. However, how mechanical signals influence macrophage polarization and contribute to bone remodeling during tooth movement remains unclear.

Mesenchymal stem cells (MSCs) can modulate macrophage polarization by secreting several bioactive and immunomodulatory factors [21–24]. MSCs are exposed to mechanical stimuli, and the function of MSCs could be modulated by mechanical stimuli [25]. Periodontal ligament stem/progenitor cells (PDLSCs), as the main MSCs in periodontal tissues, can respond to mechanical load and contribute to alveolar bone remodeling by differentiating into osteo-related cell components and promoting osteoclastogenesis [26, 27]. Previously, we have shown that periodontal ligament cells could promote inflammatory cytokines expressions in THP-1-derived macrophages under mechanical stimuli [10]. However, whether and how PDLSCs interact with macrophages under mechanical stimuli and contribute to tooth movement require further illustration.

Hydrogen sulfide (H_2_S), a signaling gas molecule, plays an important role in many physiologic and pathophysiologic processes, including maintaining mesenchymal stem cell function, limiting cardiovascular injury, regulating metabolic disorders, and mediating the inflammatory process [28–30]. H_2_S can be produced by MSCs and can regulate the MSC function to maintain bone homeostasis [28]. PDLSCs can produce H_2_S and express cystathionine-β-synthase (CBS), a H_2_S-generating enzyme [31]. Moreover, PDLSCs express H_2_S under mechanical stimulation and regulate osteoclastic activities [32]. Furthermore, H_2_S can mediate macrophage polarization during wound healing process or cardiac tissue repair after infarction [29, 33]. Therefore, we hypothesized that mechanical load-induced H_2_S production by PDLSCs modulates macrophage polarization and contributes to alveolar bone remodeling and tooth movement. Given that the signal transducer and activator of transcription 1 (STAT1) signaling pathway is critical in the activation of inflammatory M1-like macrophage polarization [19], the role of STAT1 signaling pathway during bone remodeling and tooth movement was further explored.

In this study, we used the mechanical load-induced tooth movement animal model in vivo and continuous compressive loading in vitro to test the mechanism of how mechanical load-stimulated PDLSCs produce H_2_S to promote M1 macrophage polarization and therefore contribute to alveolar bone remodeling and tooth movement.

## Methods

### Animal models with mechanical stimulation

Six- to eight-week-old male C57BL/6 mice were used in the study. All the protocols were approved by the Peking University Ethical Committee (LA2013-92). The mice were divided into four groups. Each group comprised five to six mice. Mechanical force was applied to three groups of mice for 7 days as previously described [32], and the remaining group without loading served as the control. Briefly, a nickel–titanium coil spring with 0.2-mm wire size, 1 mm in diameter, and 1 mm in length (Smart Technology, China) was bonded between the upper first molar and incisors by flowable resin (3 M, USA), which provided approximately 30 g force [34].

Vehicle (normal saline, NS), hydroxylamine (HA; 100 μg/mouse, Sigma), or GYY4137 dichloromethane complex (1 mg/mouse, Sigma) was intraperitoneally injected every other day since 1 day prior to the 7-day course of tooth movement in the three groups with mechanical loading [28]. After 7 days, the mice were sacrificed, and the maxillae were harvested. Tooth movement distance was measured from the occlusal view of the maxilla using a stereomicroscope (SWZ1000; Nikon) as previously described [9]. Briefly, the tooth movement distance was measured between the midpoint of the distal-marginal ridge of the first molar and midpoint of the mesial-marginal ridge of the second molar. The distance was measured thrice by a trained researcher who was blinded by the experimental design.

### PDLSC culture and mechanical loading

Isolation of human primary culture PDLSCs was performed as previously described [26]. The Peking University Ethical Committee has approved the protocols (PKUSSIRB-201311103), and informed consents were signed by the patients. Briefly, the periodontal ligament scraped from the root surface was digested in a mixture of 3 mg/ml type 1 collagenase (Worthington Biochem, Freehold, USA) and 4 mg/ml dispase II (Roche, Mannheim, Germany) for 1 h at 37 °C. The single-cell suspensions were then used for cell culture after passing the incubated mixture through a 70-μm strainer. The PDLSCs were identified as previously described [35] and used at the fourth passage.

When cell confluence was approximately 80%, 1 g/cm^2^ continuous compressive force was applied to the PDLSCs for 24 h [36] by using class layers and 50 mL plastic tube caps containing weighed metal balls following a modified previously described method [37] (Fig. 3a). The culture medium was collected from PDLSCs with or without loading for further experiments. H_2_S production in the culture medium from human PDLSCs was measured using a human H_2_S enzyme-linked immunosorbent assay (ELISA) kit (TSZ ELISA) according to the manufacturer’s instructions.

### Culture and treatment of THP-1-derived macrophages

THP-1 human monocytic cells (1 × 10^6^) (ATCCTIB-202) were treated with 50 ng/ml phorbol 12-myristate 13-acetate for 24 h to differentiate into macrophages.

To determine whether the production of H_2_S from load-stimulated PDLSCs can influence macrophage polarization, we cultured THP-1-derived macrophages with conditioned medium of force-treated PDLSCs with or without CBS inhibitor hydroxylamine (HA) (100 μM) [28]. Macrophages cultured with supernatant from PDLSCs without loading served as control (CS).

To investigate the mechanism of H_2_S on macrophage polarization, we treated THP-1-derived macrophages with sodium hydrosulfide (NaSH) (100 μM), HA (100 μM), and phospho-STAT1 inhibitor fludarabine (50 μM, S1491, Selleck) combined with NaSH for 24 h [28, 38]. Macrophages were pre-treated with fludarabine for 2 h. THP-1-derived macrophages were also cultured with the supernatant of force-treated PDLSCs with or without fludarabine (50 μM) for 24 h to further detect whether load-induced endogenous H_2_S production in PDLSCs influences macrophage polarization via STAT1 signaling pathway.

### Immunohistochemical staining

After sacrifice, the trimmed maxillae were fixed in 10% neutral buffered formalin for 24 h. After decalcified in ethylenediaminetetraacetic acid for 4 weeks, the tissues were then embedded by paraffin. Four-micrometer consecutive horizontal sections were obtained from the middle to apical third of the maxillary first molar, and sections from similar position of the roots were used for histological study. Immunohistochemistry was performed with a two-step detection kit (Zhongshan Golden Bridge Biotechnology, Beijing, China) as previously described [39]. The positive staining cells were counted in five different slides from each sample (*N* = 5–6). The final result came from the average of three tests.

The primary antibodies were anti-CBS (1:100, ab135626, Abcam), anti-TNF-α (1:100, ab1793, Abcam), anti-IFN-γ (1:50, sc1377, Santa Cruz), anti-IL-10 (1:200; sc-365858, Santa Cruz, Dallas, TX), and anti-CD206 (1:200, ab64693, Abcam).

### Immunofluorescence staining

Immunofluorescence staining was performed as previously described [39]. The sections were double stained with antibodies consisting anti-CD68 (1:600; MCA341GA, Serotec, UK), and anti-inducible nitric oxide synthase (iNOS) (1:100; ab-15323, Abcam), or anti-CD68 and anti-CD163 (1:100; sc-33560, Santa Cruz) to detect M1 or M2 macrophages. In addition, anti-phospho-STAT1 (Tyr701) (1:300; #9167, Cell Signaling) antibodies were used to detect the influence of H_2_S on the STAT1 signaling pathway.

The sections were then incubated with fluorescein isothiocyanate-conjugated or tetramethylrhodamine isothiocyanate-conjugated secondary antibody (1:200, Jackson Immuno Research Laboratories, West Grove, PA). Nuclei were counterstained with 4′,6-diamidino-2-phenylindole (DAPI). Confocal microscopic images were acquired using a laser scanning microscope (LSM 510, Zeiss, Jena, Germany), and the images were processed using LSM 5 Release 4.2 software. The positively stained cells were counted in five different slides from each sample (*N* = 5–6). The final result came from the average of three tests.

### Tartrate-resistant acid phosphatase (TRAP) staining

TRAP staining was conducted according to a leukocyte acid phosphatase kit (387A-1KT; Sigma, USA). Only the TRAP-positive cells (> 2 nuclei) located within the compressive PDL and the surface of the adjacent alveolar bone were considered.

### Quantitative real-time polymerase chain reaction (PCR)

Total RNA was isolated from cultured cells with TRizol reagent (Invitrogen, Carlsbad, CA) in accordance with the manufacturer’s protocol. Reverse transcription and real-time PCR were performed as previously described [39]. The sequences of primers, which were designed by Primer Premier 5.0 software and commercially synthesized, were listed as follows.

Human GAPDH sence/antisence:

5′-ATGGGGAAGGTGAAGGTCG-3′/5′-GGGGTCATTGATGGCAACAATA-3′.

Human TNF-α sence/antisence:

5′-GAGGCCAAGCCCTGGTATG-3′/5′-CGGGCCGATTGATCTCAGC-3′.

Human IL-1β sence/antisence:

5′-ATGATGGCTTATTACAGTGGCAA-3′/5′-GTCGGAGATTCGTAGCTGGA-3′.

Human DECTIN sence/antisence:

5′-GGAAGCAACACATTGGAGAATGG-3′/5′-CTTTGGTAGGAGTCACACTGTC-3′.

Human arginase-1 sence/antisence:

5′-TGGACAGACTAGGAATTGGCA-3′/5′-CCAGTCCGTCAACATCAAAACT-3′.

The amplification specificity was confirmed by the melting curve. The efficiency of PCR was confirmed by sequencing the products.

### Western blot analysis

Western blot tests were performed as previously described [10]. The primary antibodies included anti-β-actin antibody (1:10000, a-5441, Sigma), anti-iNOS (1:500, abs130136, Absin), anti-TNF-α (1:500, ab1793, Abcam), anti-arginase-1 antibody (1:1000, sc-21050, Abcam), anti-STAT1 antibody (1:1000, #9172, Cell Signaling), and anti-phospho-STAT1 antibody (1:1000, #9167, Cell Signaling). The blots were then developed by horseradish peroxidase-conjugated secondary antibodies. Finally, the blots were enhanced by chemiluminescence detection before photography. The relative density of the comparable results was measured by ImageJ 1.37v software (Wayne Rasband).

### Statistical analysis

SPSS 20.0 was used to conduct statistical analysis. Data were presented as mean ± standard deviation. The results were tested by one-way ANOVA and post hoc study was used to compare the differences between groups. Statistical significance was considered at *P* < 0.05.

## Results

### Mechanical load-induced M1-like macrophage polarization to promote alveolar bone remodeling and tooth movement depends on H_2_S production

**Fig. 1:**
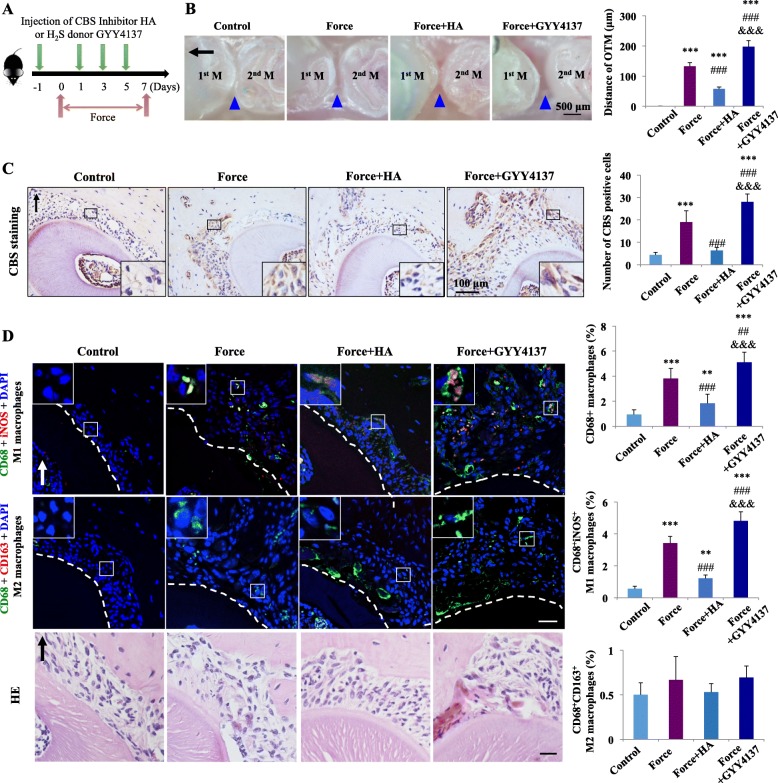
Mechanical load-induced M1 macrophage polarization during tooth movement depends on H_2_S production. **a** Schematic illustration. **b** Representative images and semiquantification analysis of the tooth movement distance in mice. Mechanical load increased the tooth movement distance, and this result was partially reversed by HA injection and enhanced by GYY4137 injection. *N* = 5–6. ***P < 0.001 versus control; ^###^*P* < 0.001 versus force; ^&&&^*P* < 0.001 versus force + HA. 1^st^ M, the first molar; 2^nd^ M, the second molar. Black arrow represents the direction of mechanical force. Blue triangles represent the tooth movement distance. Scale bar: 500 μm. **c** Representative immunohistochemical images and semiquantification analysis of cystathionine-β-synthase (CBS) expression on the compression side of distal roots. Large boxed areas show high-magnification views of the small boxed areas. *N* = 5–6. ***P < 0.001 versus control; ^###^*P* < 0.001 versus force. ^&&&^*P* < 0.001 versus force + HA. Scale bar: 100 μm. **d** Representative immunofluorescence images and the corresponding hematoxylin–eosin (HE) staining of the compression side of distal roots and semiquantification of positive cells. The changes in CD68- (green) and inducible nitric oxide synthase (iNOS)-positive (red) M1 macrophage polarization (merged yellow) and CD68- (green) and CD163-positive (red) M2 macrophage polarization (merged yellow) are presented. Dashed lines mark the root outline. Arrow represents the direction of mechanical force. Large boxed areas show high-magnification views of the small boxed areas. Scale bar: 50 μm. *N* = 5–6; the positive staining cells were counted on five different slides from each sample. The final result came from the average of three tests. **P < 0.01, ***P < 0.001 versus control. ^###^*P* < 0.001 versus force. ^&&&^*P* < 0.001 versus force + HA

To explore the influence of H_2_S on macrophage polarization under mechanical load, we blocked or enhanced the H_2_S level in mice by the systemic administration of CBS inhibitor HA or H_2_S donor GYY4137 during tooth movement (Fig. 1a). H_2_S concentration in the serum increased after GYY4137 administration and decreased after HA administration, which verified the validity of H_2_S blockage and enhancement (Supplementary Fig. 1). After force was applied for 7 days, the tooth movement distance reached 132.3 ± 12.5 μm, which decreased to 57.8 ± 6.2 μm after HA injection. GYY4137 injection could further enhance the tooth movement distance to 197.7 ± 19.8 μm (*P* < 0.001, Fig. 1b). Meanwhile, CBS expression on the compression side of periodontal tissue was elevated after loading (*P* < 0.001), and it was suppressed after HA injection (*P* < 0.001) and enhanced after GYY4137 injection (*P* < 0.001) compared with the force group (Fig. 1c).

Concomitantly, immunofluorescence staining showed that CD68-positive macrophages accumulated on the compression side of periodontal ligament after force was applied for 7 days. HA injection significantly decreased the percentage of macrophages, and GYY4137 injection resulted in their increase compared with the force group (*P* < 0.001). The percentage of CD68^+^iNOS^+^ M1-like macrophages increased to 3.39% ± 0.43% after force was applied compared with 0.6% ± 0.13% in the control group. HA injection significantly decreased the percentage of M1-like macrophages to 1.3% ± 0.3% compared with the force group (*P* < 0.001). The percentage of M1-like macrophages further increased to 4.85% ± 0.52% after GYY4137 injection (*P* < 0.001; Fig. 1d). However, CD68^+^CD163^+^ M2-like macrophages were hardly detected (Fig. 1d).

**Fig. 2:**
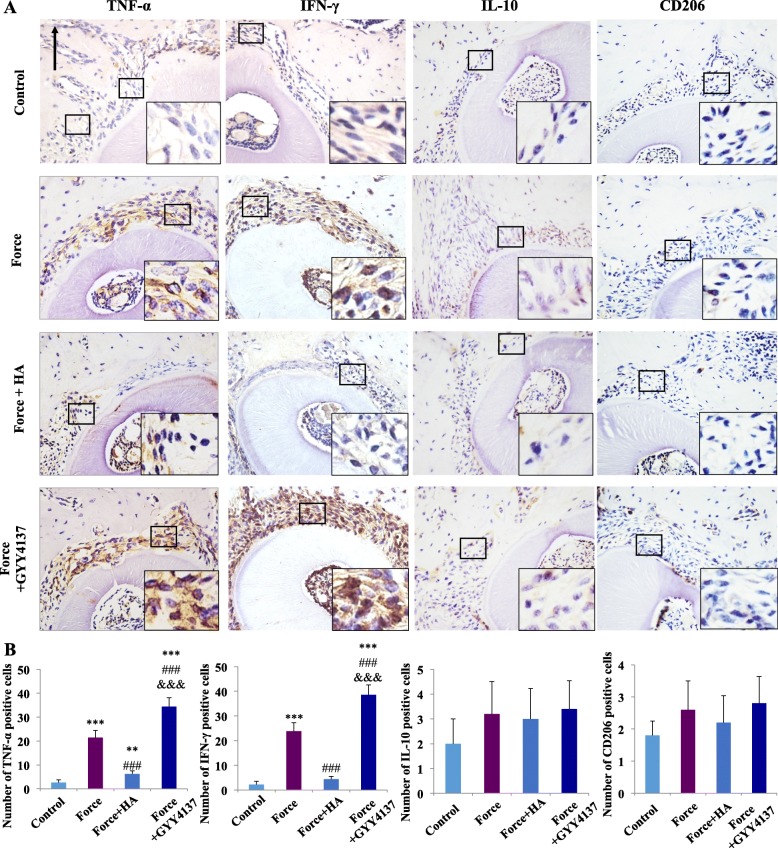
Mechanical load-induced expression of M1/M2-associated markers depends on H_2_S. **a** Representative immunohistochemical images of the compression side of distal roots. Expressions of M1-associated cytokines TNF-α and IFN-γ were upregulated after force was applied; the expressions decreased after HA injection and further enhanced after GYY4137 injection. Conversely, the expressions of M2-associated markers IL-10 and CD206 were hardly changed. Large boxed areas show high-magnification views of the small boxed areas. Arrow represents the direction of mechanical force. Scale bars: 100 μm. **b** Semiquantification of positive cells. *N* = 5–6; positive staining cells were counted on five different slides from each sample. The final result came from the average of three tests. **P < 0.01, ***P < 0.001 versus control. ^###^*P* < 0.001 versus force. ^&&&^*P* < 0.001 versus force + HA

The contribution of H_2_S to the expression levels of macrophage-associated markers on the compression side of periodontal tissues was also investigated. The expressions of M1-macrophage-associated pro-inflammatory cytokines, TNF-α and IFN-γ, were upregulated after force was applied for 7 days (*P* < 0.001). HA administration significantly decreased the expression levels of TNF-α and IFN-γ, whereas GYY4137 administration resulted in their increase (*P* < 0.001, Fig. 2). Correspondingly, the upregulated expression of TRAP^+^ osteoclasts in the force group was further suppressed by HA administration and enhanced by GYY4137 administration (*P* < 0.001, Supplementary Fig. 2), which was consistent with our previous studies [32].

However, the expression of M2-macrophage-associated markers IL-10 and CD206 showed no change in the four groups (Fig. 2). Specifically, the administration of HA or GYY4137 without loading cause no change in the number of CD68-positive macrophages or TRAP-positive osteoclasts (Supplementary Fig.3). These data indicate that both endogenous H_2_S level and exogenous H_2_S administration regulated M1-like macrophage polarization and the subsequent alveolar bone remodeling and tooth movement process under mechanical stimuli.

### Mechanical load-stimulated PDLSCs produce H_2_S to promote M1 macrophage polarization in vitro

**Fig. 3:**
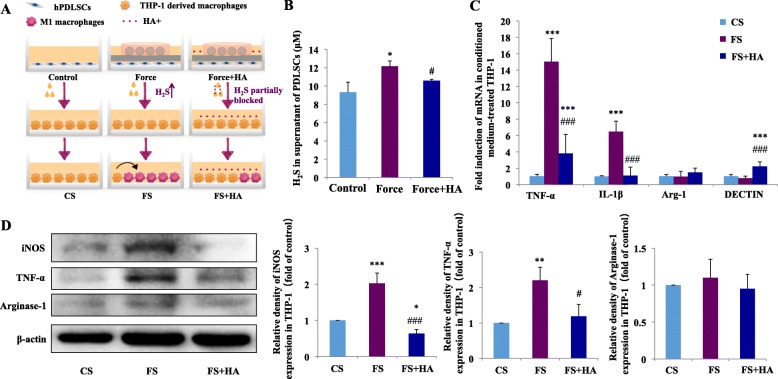
H_2_S production in mechanical load-stimulated periodontal ligament stem cells (PDLSCs) and the influence on macrophage polarization. **a** Schematic illustration. **b** Concentration of H_2_S in the supernatant of PDLSCs was upregulated after mechanical loading and downregulated by additional HA treatment with loaded conditioned medium. *N* = 3, *P < 0.05 versus control, ^#^*P *< 0.05 versus force. **c** Relative mRNA expression of M1/M2-related genes. The mRNA expressions of M1 markers TNF-α and IL-1β of THP-1-derived macrophages were upregulated in FS group and downregulated in FS + HA group compared with the FS group, although the expression of TNF-α was still upregulated compared with the CS group. The mRNA expression of arginase-1 shows no change, and the expression of DECTIN was upregulated in the FS + HA group. *N* = 3, ***P < 0.001 versus CS. ^###^*P* < 0.001 versus FS. CS: control supernatant; FS: force-treated conditioned medium. FS + HA: force-treated conditioned medium with CBS inhibitor HA. **d** Western blot of iNOS, TNF-α and arginase-1. iNOS and TNF-α expressions of THP-1-induced macrophages were upregulated after incubated with the supernatant of force-treated PDLSCs and decreased after incubated with the supernatant of force-treated PDLSCs with HA application. Meanwhile, no changes were observed in arginase-1 expression. Beta-actin served as the internal control for equal loading. Data represent three independent experiments. *P < 0.05, **P < 0.01, ***P < 0.001 versus CS. ^#^*P* < 0.05, ^###^*P* < 0.001 versus FS

Mechanical load can upregulate H_2_S expression in PDLSCs [32]. H_2_S concentration in the supernatant of PDLSCs was significantly upregulated after compressive loading and suppressed by the additional treatment of HA with compressive loading (Fig. 3b). Whether H_2_S expression in force-treated PDLSCs could promote THP-1-derived macrophages to polarize toward M1 phenotype was further explored in this study. To this end, THP-1-derived macrophages were cultured with the supernatants from force-treated primary PDLSCs with or without CBS inhibitor HA (FS + HA or FS). Macrophages cultured with supernatant from PDLSCs without loading served as the control (CS) (Fig. 3a).

The mRNA expression levels of M1 markers TNF-α and IL-1β of THP-1-derived macrophages were dramatically upregulated in the FS group (*P* < 0.001 versus CS) and downregulated in the FS + HA group (*P* < 0.001 versus FS), although the expression of TNF-α was still upregulated compared with the CS group (*P* < 0.001). Conversely, the mRNA expression of M2 marker arginase-1 showed no change, and the expression of DECTIN was upregulated in the FS + HA group (*P* < 0.001) (Fig. 3c).

Western blot analysis showed that the protein expression of M1-associated markers iNOS and TNF-α increased in the FS group and decreased in the FS + HA group. However, no changes were observed in the expression of arginase-1 (Fig. 3d). Specifically, we applied HA with PDLSC-conditioned medium without loading to treat THP-1 macrophages, and no significant changes in the expressions of TNF-α and arginase-1 were detected (Supplementary Fig. 4A). These findings suggest that upregulated H_2_S production in mechanical load-stimulated PDLSCs can promote THP-1-derived macrophages to polarize into M1 phenotype.

### Mechanical load-induced H_2_S contributes to M1 macrophage polarization via the STAT1 signaling pathway

**Fig. 4:**
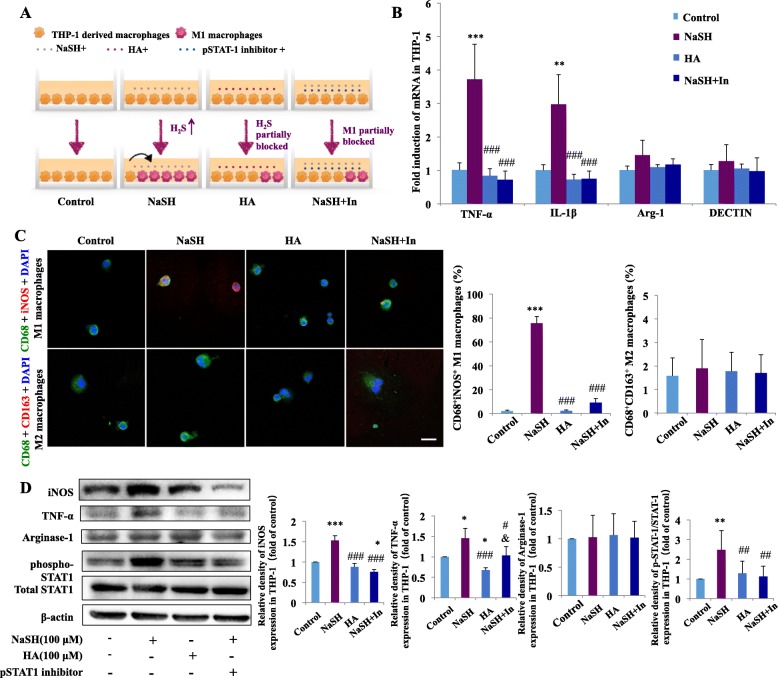
Changes in STAT1 pathway in H_2_S induced macrophage polarization. **a** Schematic illustration. **b** Relative mRNA expressions of M1/M2-related genes. The mRNA expressions of TNF-α and IL-1β of THP-1-derived macrophages were upregulated in the NaSH group and downregulated in the HA group and NaSH + pSTAT1 inhibitor groups compared with the NaSH group. The mRNA expressions of arginase-1 and DECTIN showed no changes. *N* = 3, **P < 0.01, ***P < 0.001 versus control. ^###^*P* < 0.001 versus NaSH. **c** Representative immunocytochemical images of THP-1 derived macrophages. CD68-positive (green) and iNOS-positive (red) M1 macrophage polarization increased after NaSH treatment, which decreased significantly after HA or NaSH + phospho-STAT1 inhibitor treatment compared with the NaSH group. Meanwhile, CD68-positive (green) and CD163-positive (red) M2 macrophage polarization exhibited no change in the four groups. Scale bar: 50 μm. ***P < 0.001 versus control, ^###^*P* < 0.001 versus NaSH. **d** Western blot results and semi-quantifications of iNOS, TNF-α, arginase-1, phosphorylation of STAT1, and total STAT1 expression in macrophages. iNOS and TNF-α expressions were upregulated after NaSH application and were decreased by HA application or additional administration of pSTAT1 inhibitor with NaSH. The proportion of pSTAT1/STAT1 was upregulated after NaSH application and was partially reversed by HA application or by additional administration of pSTAT1 inhibitor. Beta-actin served as the internal control for equal loading. Data represent three independent experiments. *P < 0.05, **P < 0.01, ***P < 0.001 versus control, ^#^*P* < 0.05, ^##^*P* < 0.01, ^###^*P* < 0.001 versus NaSH, ^&^*P* < 0.05 versus HA

We further examined the mechanism of how exogenous H_2_S and endogenous H_2_S productions in mechanical load-stimulated PDLSCs promote M1 macrophage polarization. The STAT1 signaling pathway plays a critical role in M1 macrophage polarization [19]. To detect whether exogenous H_2_S promotes THP-1-derived macrophages to polarize toward M1 phenotype via the STAT1 signaling pathway, we treated THP-1-derived macrophages with H_2_S donor NaSH and CBS inhibitor HA and used NaSH + phospho-STAT1 inhibitor fludarabine to test the function of STAT1 pathway (Fig. 4a). The level of H_2_S in the culture medium of THP-1-derived macrophages was enhanced by NaSH treatment and decreased after HA treatment (Supplementary Fig. 5).

The mRNA expression levels of the M1 markers TNF-α and IL-1β in THP-1-derived macrophages were upregulated after NaSH treatment (*P* < 0.001 and *P* < 0.01, respectively), which were reversed after treatment with HA and NaSH + phospho-STAT1 inhibitor (*P* < 0.001 versus NaSH). Conversely, the mRNA expression levels of M2 markers arginase-1 and DECTIN showed no change (Fig. 4b).

Immunocytochemical analyses showed that the proportion of CD68^+^iNOS^+^ M1 macrophages increased significantly after NaSH treatment (*P* < 0.001) and decreased significantly after HA or NaSH + phospho-STAT1 inhibitor treatment (*P* < 0.001 versus NaSH). Meanwhile, the proportion of CD68^+^CD163^+^ M2 macrophages showed no changes (Fig. 4c). Western blot analysis showed that the expressions of iNOS and TNF-α were significantly upregulated after NaSH treatment (*P* < 0.001 and *P* < 0.05, respectively) and downregulated after HA treatment (*P* < 0.05). The upregulation of iNOS and TNF-α was partially reversed by the additional administration of phospho-STAT1 inhibitor with NaSH (*P* < 0.001 and *P* < 0.05 versus NaSH, respectively). Meanwhile, the proportion of phospho-STAT1/STAT1 was also upregulated after NaSH treatment (*P* < 0.01) and was partially reversed by HA treatment or by additional administration of phospho-STAT1 inhibitor (*P* < 0.01 versus NaSH). However, no changes were observed in arginase-1 expression (Fig. 4d). These results indicate that macrophages were polarized toward M1 phenotype after treatment with NaSH.

**Fig. 5:**
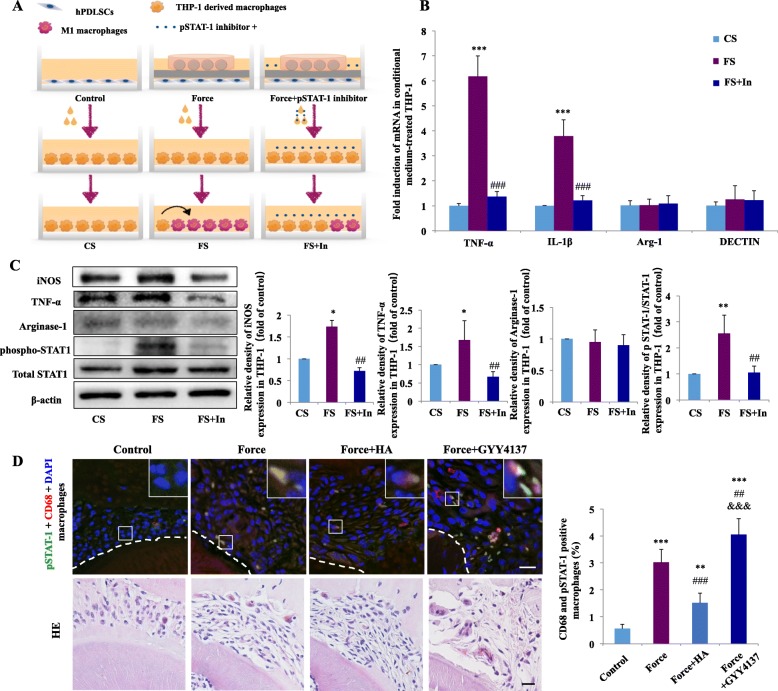
Changes in STAT1 pathway in macrophages stimulated by mechanical load-induced H_2_S production. **a** Schematic illustration. **b** Relative mRNA expression of M1/M2-related genes. The mRNA expressions of M1 markers TNF-α and IL-1β of THP-1-derived macrophages were upregulated in the FS group and downregulated in the FS + In group compared with the FS group. The mRNA expressions of arginase-1 and DECTIN showed no changes. *N* = 3, ***P < 0.001 versus CS. ^###^*P* < 0.001 versus FS. CS: control supernatant; FS: force-treated conditioned medium. FS + In: force-treated conditioned medium with pSTAT1 inhibitor. **c** Western blot results of iNOS, TNF-α, arginase-1, phosphorylation of STAT1, and total STAT1 expressions in macrophages incubated with different conditioned medium. iNOS, TNF-α expressions, and the proportion of pSTAT1/STAT1 in macrophages were upregulated after incubated with the force-treated conditioned medium of PDLSCs, which were partially reversed by incubation with the force-treated conditioned medium with pSTAT1 inhibitor. Beta-actin served as the internal control for equal loading. Data represent three independent experiments. **P < 0.01 versus CS, ^##^*P* < 0.01 versus FS. **d** Representative immunofluorescence images and the corresponding hematoxylin–eosin (HE) staining of the compression side of distal roots and semiquantification of double-stained positive cells. Mechanical load increased the proportion of CD68-positive (red) and pSTAT1-positive (green) macrophages (merged yellow), which decreased after HA injection and enhanced after GYY4137 injection. Dashed lines mark the root outline. Scale bar: 50 μm. Arrow represents the direction of force. Large boxed areas show high-magnification views of the small boxed areas. *N* = 5–6; **P < 0.01, ***P < 0.001 versus control. ^##^*P* < 0.01, ^###^*P* < 0.001 versus force. ^&&&^*P* < 0.001 versus force + HA

To confirm whether load-induced endogenous H_2_S production in PDLSCs promotes THP-1-derived macrophages to polarize toward M1 phenotype via the STAT1 signaling pathway, we cultured THP-1-derived macrophages with the supernatants from force-treated PDLSCs with or without phospho-STAT1 inhibitor fludarabine (FS + In or FS). Macrophages cultured with supernatant from PDLSCs without loading served as the control (CS) (Fig. 5a).

Real-time PCR showed that the mRNA expressions of TNF-α and IL-1β in THP-1-derived macrophages were upregulated after incubation with the force-treated conditioned medium of PDLSCs (*P* < 0.001 versus CS) and downregulated after incubation with the force-treated conditioned medium with fludarabine (*P* < 0.001 and *P* < 0.01 versus FS). Conversely, the mRNA expressions of arginase-1 and DECTIN remained unchanged (Fig. 5b).

The western blot results showed that the protein expression levels of iNOS and TNF-α and the proportion of phospho-STAT1/STAT1 were significantly upregulated after incubation with the force-treated conditioned medium of PDLSCs (*P* < 0.01). These enhancing effects were partially reversed by the incubation with the force-treated conditioned medium with fludarabine (*P* < 0.01 versus LM). Meanwhile, no changes were observed in arginase-1 expression (Fig. 5c). Specifically, we applied fludarabine with PDLSC-conditioned medium without loading to treat THP-1 macrophages, and no significant changes were detected in the expressions of TNF-α and arginase-1 (Supplementary Fig. 4B). These data indicate that exogenous H_2_S and endogenous H_2_S production in force-treated PDLSCs could promote M1 macrophage polarization by activating the STAT1 signaling pathway.

To further confirm the involvement of STAT1 pathway in the macrophages in vivo, we performed immunofluorescence staining to show the co-expression of STAT1 with CD68 on the compression side of the periodontal tissues. CD68^+^phospho-STAT1^+^ macrophages accumulated on the compression side of periodontal tissues during tooth movement, with positive cells of 3.03% ± 0.48% in the force group compared with 0.56% ± 0.16% in the control group (*P* < 0.001). The percentage of CD68^+^phospho-STAT1^+^ macrophages significantly decreased to 1.52% ± 0.36% after HA application compared with the force group (*P* < 0.001) but remained higher than that in the control group (*P* < 0.01). Moreover, the application of GYY4137 dramatically increased the percentage of CD68^+^ phospho-STAT1^+^ macrophages to 4.05% ± 0.59% (*P* < 0.001, Fig. 5d).

**Fig. 6:**
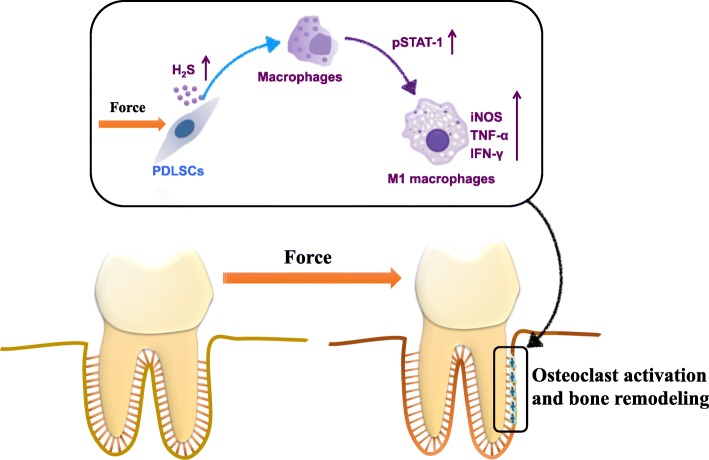
Scheme. Mechanical load-stimulated PDLSCs enhance H_2_S production to promote M1 macrophage polarization under mechanical stimuli via the activation of the STAT1 signaling pathway, which leads to bone remodeling process during tooth movement

Taken together, our results indicate that mechanical load-stimulated PDLSCs could enhance H_2_S production to promote M1 macrophage polarization during tooth movement by activating the STAT1 signaling pathway (Fig. 6).

## Discussion

The conversion of mechanical stimulation into biochemical reactions is essential for intercellular communication, and this process may control bone remodeling in mammals [6, 7]. Tooth movement is a unique aseptic inflammation-associated bone remodeling process induced by mechanical stimulation [8, 13]. During this process, PDLSCs and macrophages are the main cellular components within the microenvironment of periodontal tissues. PDLSCs are the unique MSCs that can respond to mechanical stimuli and express multiple cytokines, chemokines, and other inflammatory elements to influence bone remodeling [27, 32, 40]. The detailed mechanism of how PDLSCs contribute to bone remodeling process under mechanical stimuli has not been illustrated. Macrophages, especially M1 phenotype, play critical roles in mechanical load-induced bone remodeling and tooth movement [10]. However, whether and how PDLSCs interact with macrophages under mechanical stimulation remain largely unknown. In this study, we showed a novel mechanism in which PDLSCs produce H_2_S to modulate M1 macrophage polarization to promote bone remodeling and tooth movement under mechanical stimuli. First, mechanical load promoted M1 macrophage polarization on the compression side of periodontal ligament during tooth movement, accompanied by increased CBS expression. The enhancement or blockage of H_2_S led to the increased or repressed of the expression of M1 macrophages and distance of tooth movement. Second, mechanical stimulation enhanced H_2_S production in PDLSCs, which increased the expression of M1-associated cytokines in macrophages. These effects could be blocked by the administration of CBS inhibitor HA. Finally, we confirmed that mechanical load-induced H_2_S promoted M1 macrophage polarization via the STAT1 signaling pathway. These data suggest that mechanical load-stimulated PDLSCs produced H_2_S to polarize macrophages toward M1 phenotype via the STAT1 signaling pathway, thus contributing to alveolar bone remodeling and tooth movement.

Although M1 macrophage polarization is considered important in mechanical load-induced bone remodeling during tooth movement [10], how M1 macrophages polarize under mechanical stimuli remains poorly understood. The phenotypes of macrophages change depending on different environmental elements [16, 17]. Inflammatory-associated elements, such as IFN-γ or lipopolysaccharides, cause the activation of M1 polarization, whereas IL-4 or IL-13 can induce the activation of M2 polarization [17–19]. Here, we observed that the changes in H_2_S level under mechanical stimuli and exogenous H_2_S application or inhibition could modulate the number of M1-like macrophages in periodontal tissues and influence bone remodeling and tooth movement. Further in vitro experiments showed that H_2_S production was upregulated in loaded PDLSCs, which promoted macrophages to polarize into M1 phenotypes. Our study uncovered a novel finding indicating that mechanical stimuli could modulate M1 macrophage polarization by inducing H_2_S production in PDLSCs, therefore contributing to alveolar bone remodeling and tooth movement.

Raising evidence has shown that H_2_S serves as a gasotransmitter that regulates various signaling pathways [41]. H_2_S can also mediate macrophage polarization during wound healing or cardiac tissue repair after infarction, probably through mitochondrial biogenesis, fatty acid oxidation, and the nuclear factor-κB signaling pathway [29, 33]. However, the molecular mechanism of how H_2_S regulates the differentiation of M1 macrophages under mechanical stimulation remains unclear. In the present study, both load-induced endogenous H_2_S production in PDLSCs and the exogenous administration of H_2_S could promote M1 macrophage polarization via the STAT1 signaling pathway, which is a critical signaling pathway in M1 macrophage polarization [19]. Our results uncovered the novel mechanism of H_2_S regulation on M1 macrophage polarization via the STAT1 signaling pathway under mechanical stimuli, which differs from the previously reported biochemical regulatory mechanism.

The activation of STAT1 is related to various signaling pathways, including the IFN-α/β and IFN-γ pathways [5, 12, 13]. Meanwhile, H_2_S serves as a gasotransmitter to regulate a variety of signaling pathways. H_2_S can control cellular Ca^2+^ levels and affect multiple transient receptor potential calcium channels [42]. Whether H_2_S activates STAT1 through regulating the extracellular messenger molecule binding to specific cell surface receptors or by regulating ionic channels needs to be further explored.

The interactions between MSCs and immune cells are important in bone and tissue regeneration and the healing of multiple pathological processes [43–45]. MSCs can modulate macrophage polarization by reducing inflammation and mediating tissue repair through the secretion of extracellular vesicles, exosomes, or cytokines such as transforming growth factor-β [21–24]. PDLSCs are unique MSCs that constantly receive mechanical stimulation. These cells are sensitive to mechanical stimulation both in vitro and in vivo and can express high levels of inflammatory cytokines, chemokines, and gas molecular H_2_S under mechanical stimuli [27, 32, 40]. However, how PDLSCs interact with immune cells and contribute to bone remodeling under mechanical stimuli still needs further illustration. Our study revealed that H_2_S concentration in the supernatant of loaded PDLSCs was upregulated by approximately 1.3-fold, which increased the expressions of M1-associated cytokines in macrophages. The increased H_2_S production and M1 cytokine expression could be partially reversed by the additional application of HA. These results confirm that PDLSCs can promote M1 macrophage polarization by producing the gaseous signaling molecule H_2_S under mechanical stimulation, thus shedding light on the novel mechanism of how MSCs regulate macrophage polarization under mechanical stimulation. Nevertheless, further experiments should be conducted to explore whether other cytokines produced by PDLSCs would influence macrophage polarization under mechanical stimuli.

Regarding the relationship between macrophage polarization and alveolar bone resorption, macrophages and osteoclasts are derived from myeloid precursor cells of hematopoietic origin [46]. Previous research has shown that M1 macrophage-related cytokines such as TNF-α, IL-6, and IL-1β could induce osteoclastogenesis. These cytokines could directly increase osteoclast precursor numbers and differentiation and indirectly promote osteoblasts and other stromal cells to increase RANKL production [47, 48]. On the contrary, M2 macrophage-related cytokines, such as IL-4 and IL-10, can inhibit osteoclastogenesis [47]. On the compressive side of periodontal ligament during tooth movement, M1 macrophages accumulated after mechanical stimulation, which contributed to the expression of inflammatory cytokines. Furthermore, PDLSCs can increase the expressions of TNF-α, IL-1β, and the RANKL/osteoprotegrin ratio under mechanical loading [9, 49]. Therefore, the accumulation of M1 macrophage polarization on the compression side of periodontal tissues during mechanical load-induced tooth movement may lead to bone resorption. Nevertheless, the underlying molecular mechanisms of the interaction between macrophages and osteoclasts still require elucidation. Macrophages and osteoclasts are assumed to be competing differentiation outcomes from myeloid progenitors. Several nuclear receptors/transcription factors could promote osteoclast differentiation but suppress macrophage activation [50]. Further studies should be conducted to illustrate the relationship between macrophages and osteoclasts.

## Conclusions

In conclusion, mechanical load-induced H_2_S production by PDLSCs polarizes macrophages toward M1 phenotype via the STAT1 signaling pathway, which contributes to bone remodeling and tooth movement. These results aid in elucidating the mechanism of how PDLSCs influence macrophage polarization and contribute to bone remodeling under mechanical stimuli. The findings also provide new insights indicating that appropriate H_2_S supplementation may accelerate tooth movement.

## Supplementary information

Additional file 1: Figure S1. H_2_S concentration in the serum of mice. H_2_S concentration in the serum increased after GYY4137 administration and decreased after HA administration. *P < 0.05 versus control. ^#^*P *< 0.05 versus GYY4137.

Additional file 2: Figure S2. Representative tartrate-resistant acid phosphatase (TRAP) staining images of the compression side of distal roots. (A) The number of TRAP-positive osteoclasts was upregulated after force was applied, which decreased after HA administration and further enhanced after GYY4137 administration. Large boxed areas show high-magnification views of the small boxed areas. Arrow represents the direction of force. Scale bars: 100 μm. (B) Semiquantification of positive cells. *N* = 5–6; the positive staining cells were counted in five different slides from each sample. The final result came from the average of three tests. ***P < 0.001 versus control. ^###^P < 0.001 versus force. ^&&&^P < 0.001 versus force + HA.

Additional file 3: Figure S3. Representative immunohistochemical and tartrate-resistant acid phosphatase (TRAP) staining images of different treatments in mice without force application. (A) No significant changes of the number of TRAP positive osteoclasts and the expressions of CD68 were detected in the control group and groups with HA or GYY4137 application. Data showed the compression side of distal roots. Large boxed areas show high-magnification views of the small boxed areas. Arrow represents the direction of force. Scale bars: 100 μm. (B) Semiquantification of positive cells. *N* = 5; the positive staining cells were counted in five different slides from each sample. The final result came from the average of three tests.

Additional file 4: Figure S4. Western blot results of THP-1-derived macrophages. (A) No significant changes were observed in TNF-α and arginase-1 expressions in THP-1-induced macrophages after incubated with the supernatant of PDLSCs with or without HA application. (B) No significant changes were observed in TNF-α and arginase-1 expressions in THP-1-derived macrophages after incubated with the supernatant of PDLSCs with or without pSTAT1 inhibitor application. Beta-actin served as the internal control for equal loading. Data represent three independent experiments.

Additional file 5: Figure S5. H_2_S level in the supernatant of THP-1-derived macrophages. The level of H_2_S in the culture medium of THP-1-derived macrophages was enhanced or decreased after treated with NaSH or HA. *P < 0.05 versus control. ^##^*P* < 0.01 versus NaSH.

## Abbreviations

CBS: Cystathionine-β-synthase
HA: Hydroxylamine
H_2_S: Hydrogen sulfide
IFN-γ: Interferon-γ
MSCs: Mesenchymal stem cells
NaSH: Sodium hydrosulfide
NO: Nitric oxide
PDLSCs: Periodontal ligament stem cells
STAT-1: Signal transducer and activators of transcription 1
TNF-α: Tumor necrosis factor
