# Supplementary figures and images for: Mechanical load-induced H2S production by periodontal ligament stem cells activates M1 macrophages to promote bone remodeling and tooth movement via STAT1
Source: Stem Cell Res Ther. 2020 Mar 13;11:112. doi: 10.1186/s13287-020-01607-9 (PMC7071778; doi:10.1186/s13287-020-01607-9)

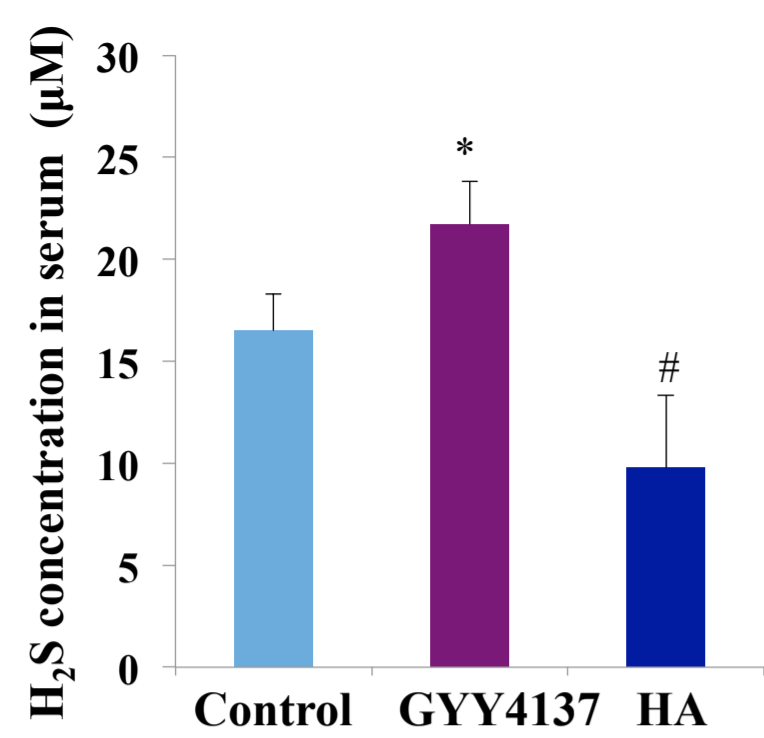

Supplement: Supplementary file 1 — Figure S1. H2S concentration in the serum of mice. H2S concentration in the serum increased after GYY4137 administration and decreased after HA administration. *P < 0.05 versus control. #P < 0.05 versus GYY4137. [file 13287_2020_1607_MOESM1_ESM.pdf]

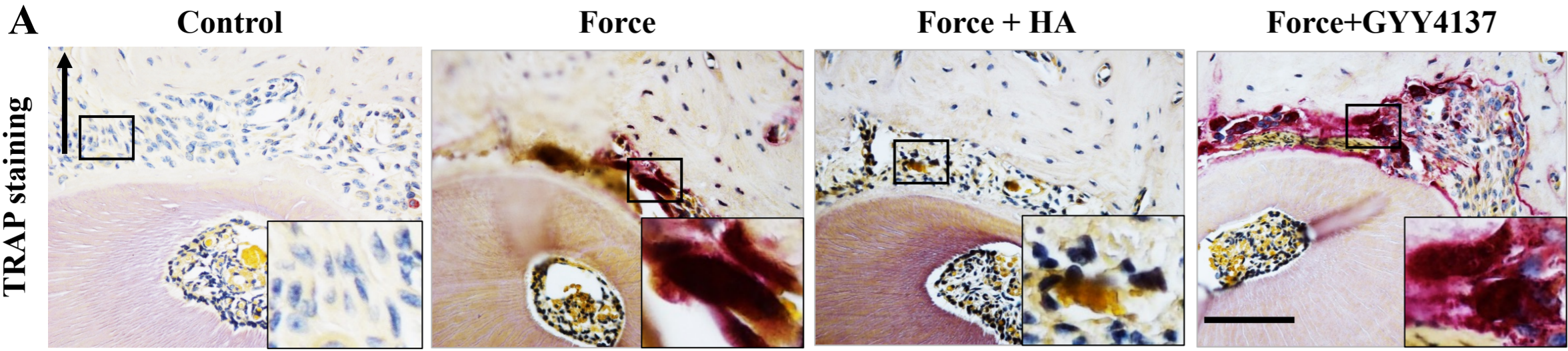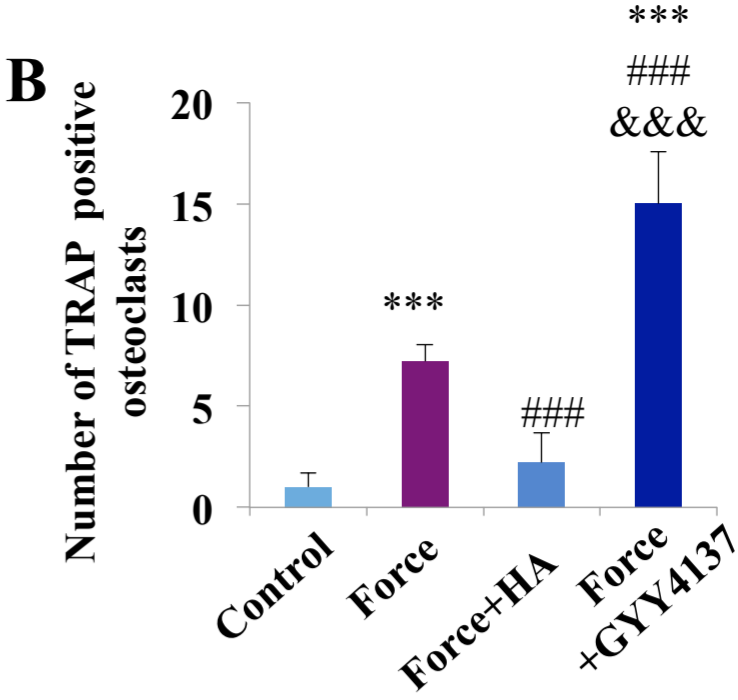

Supplement: Supplementary file 2 — Figure S2. Representative tartrate-resistant acid phosphatase (TRAP) staining images of the compression side of distal roots. (A) The number of TRAP-positive osteoclasts was upregulated after force was applied, which decreased after HA administration and further enhanced after GYY4137 administration. Large boxed areas show high-magnification views of the small boxed areas. Arrow represents the direction of force. Scale bars: 100 μm. (B) Semiquantification of positive cells. N = 5–6; the positive staining cells were counted in five different slides from each sample. The final result came from the average of three tests. ***P < 0.001 versus control. ###P < 0.001 versus force. &&&P < 0.001 versus force + HA. [file 13287_2020_1607_MOESM2_ESM.pdf]

A

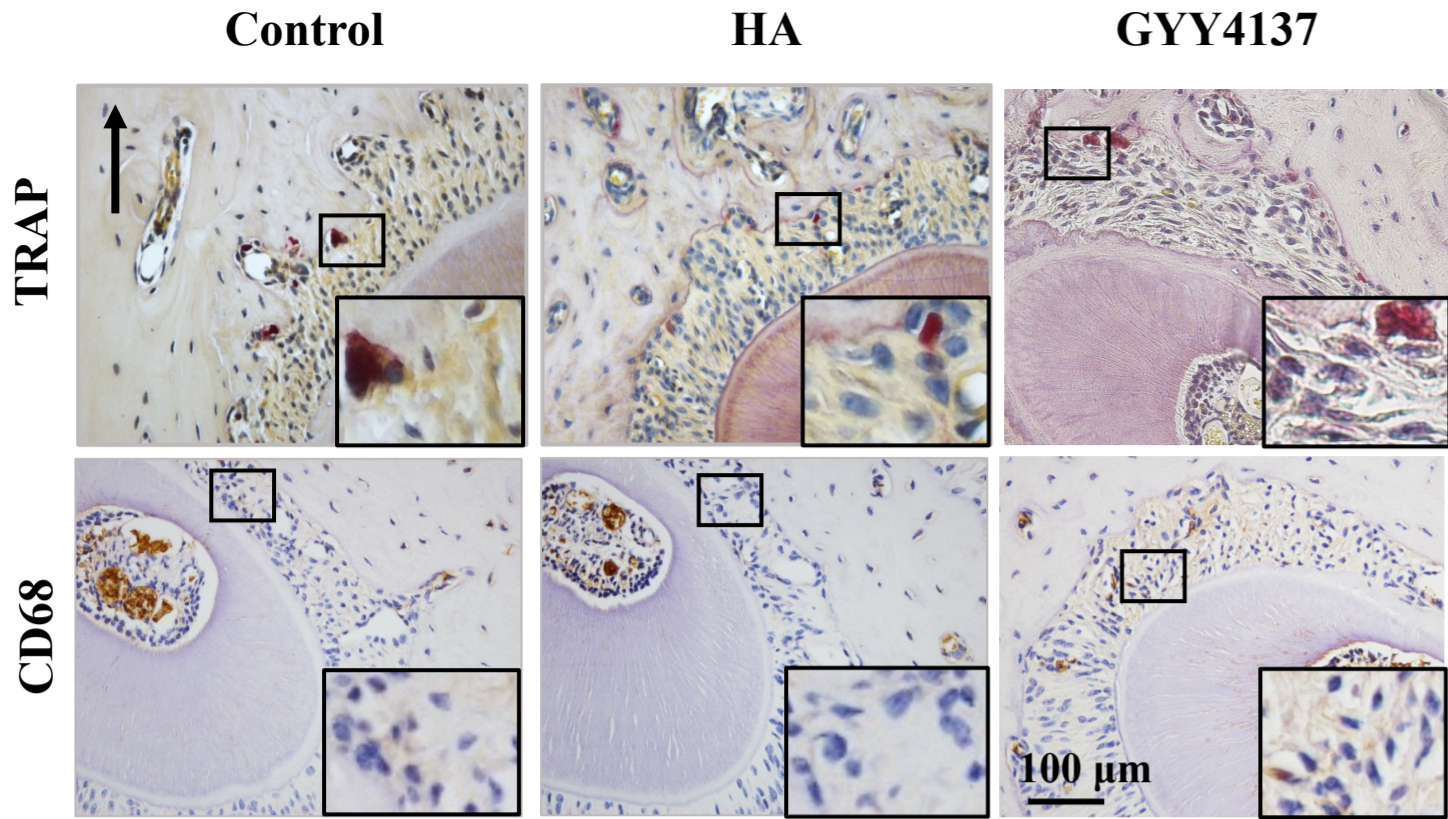

B

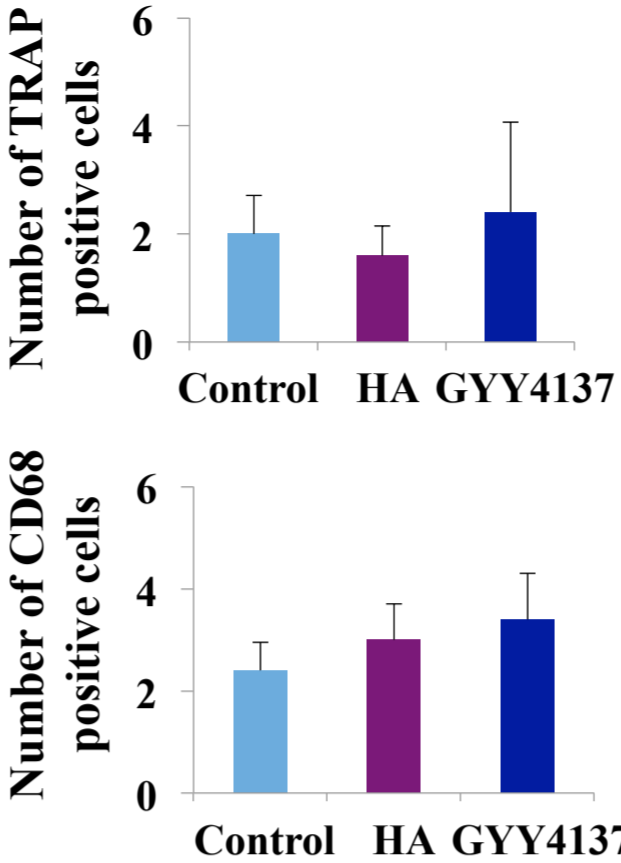

Supplement: Supplementary file 3 — Figure S3. Representative immunohistochemical and tartrate-resistant acid phosphatase (TRAP) staining images of different treatments in mice without force application. (A) No significant changes of the number of TRAP positive osteoclasts and the expressions of CD68 were detected in the control group and groups with HA or GYY4137 application. Data showed the compression side of distal roots. Large boxed areas show high-magnification views of the small boxed areas. Arrow represents the direction of force. Scale bars: 100 μm. (B) Semiquantification of positive cells. N = 5; the positive staining cells were counted in five different slides from each sample. The final result came from the average of three tests. [file 13287_2020_1607_MOESM3_ESM.pdf]

**A**

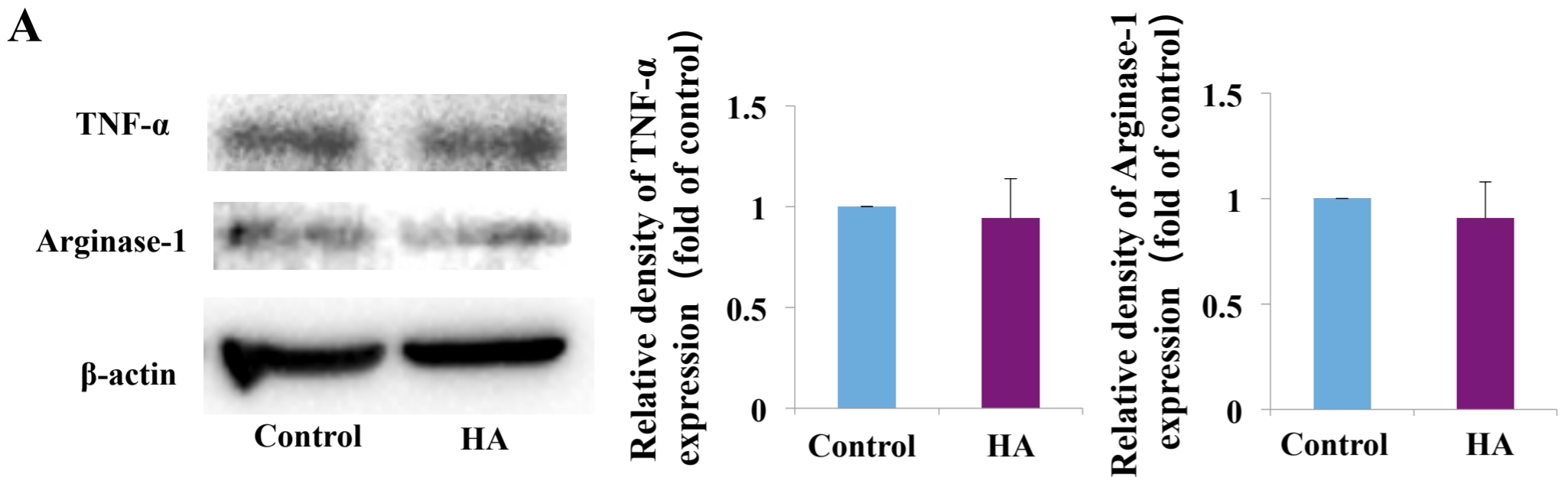

**B**

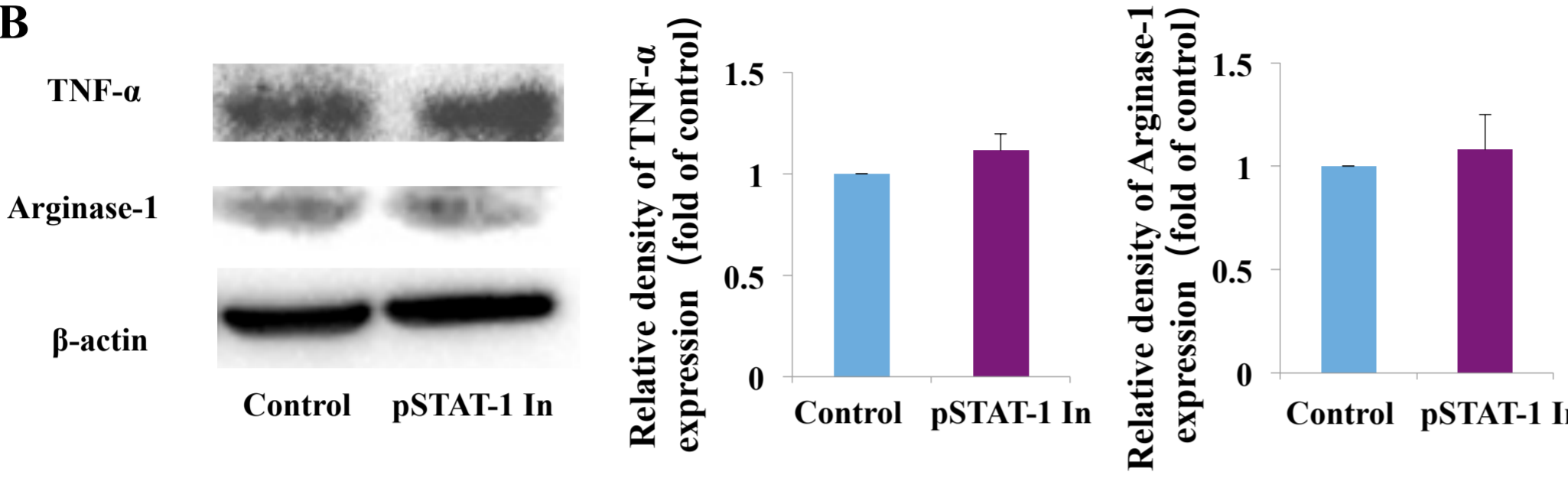

Supplement: Supplementary file 4 — Figure S4. Western blot results of THP-1-derived macrophages. (A) No significant changes were observed in TNF-α and arginase-1 expressions in THP-1-induced macrophages after incubated with the supernatant of PDLSCs with or without HA application. (B) No significant changes were observed in TNF-α and arginase-1 expressions in THP-1-derived macrophages after incubated with the supernatant of PDLSCs with or without pSTAT1 inhibitor application. Beta-actin served as the internal control for equal loading. Data represent three independent experiments. [file 13287_2020_1607_MOESM4_ESM.pdf]

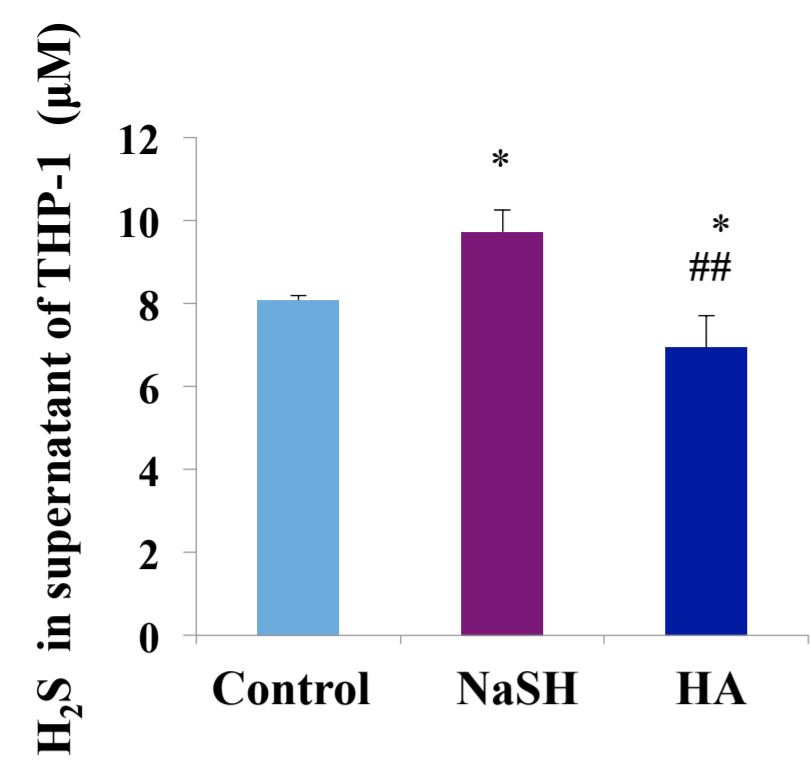

Supplement: Supplementary file 5 — Figure S5. H2S level in the supernatant of THP-1-derived macrophages. The level of H2S in the culture medium of THP-1-derived macrophages was enhanced or decreased after treated with NaSH or HA. *P < 0.05 versus control. ##P < 0.01 versus NaSH. [file 13287_2020_1607_MOESM5_ESM.pdf]
